# Bidirectional Modulation of Numerical Magnitude

**DOI:** 10.1093/cercor/bhv344

**Published:** 2016-02-14

**Authors:** Qadeer Arshad, Yuliya Nigmatullina, Ramil Nigmatullin, Paladd Asavarut, Usman Goga, Sarah Khan, Kaija Sander, Shuaib Siddiqui, R. E. Roberts, Roi Cohen Kadosh, Adolfo M. Bronstein, Paresh A. Malhotra

**Affiliations:** 1 Division of Brain Sciences, Imperial College London, London W6 8RF, UK; 2 Institut für Quantenphysik and Centre for Integrated Quantum Science and Technology (IQST), Albert Einstein Allell, Universität Ulm, Ulm D-89069, Germany; 3 Department of Experimental Psychology, Oxford University, Oxford 0X1 3UD, UK

**Keywords:** dynamic interhemispheric competition, mental number line, numerical magnitude, vestibular cognition, VOR

## Abstract

Numerical cognition is critical for modern life; however, the precise neural mechanisms underpinning numerical magnitude allocation in humans remain obscure. Based upon previous reports demonstrating the close behavioral and neuro-anatomical relationship between number allocation and spatial attention, we hypothesized that these systems would be subject to similar control mechanisms, namely dynamic interhemispheric competition. We employed a physiological paradigm, combining visual and vestibular stimulation, to induce interhemispheric conflict and subsequent unihemispheric inhibition, as confirmed by transcranial direct current stimulation (tDCS). This allowed us to demonstrate the first systematic bidirectional modulation of numerical magnitude toward either higher or lower numbers, independently of either eye movements or spatial attention mediated biases. We incorporated both our findings and those from the most widely accepted theoretical framework for numerical cognition to present a novel unifying computational model that describes how numerical magnitude allocation is subject to dynamic interhemispheric competition. That is, numerical allocation is continually updated in a contextual manner based upon relative magnitude, with the right hemisphere responsible for smaller magnitudes and the left hemisphere for larger magnitudes.

## Introduction

It is postulated that for cultural innovations such as numbers, the brain co-opts evolutionarily older and multifunctional cortical circuits (Hubbard et al. 2005; Dehaene and Cohen 2007), particularly invoking fronto-parietal networks, which are repeatedly implicated for the allocation of spatial attention (Corbetta and Shulman 2002), eye movement control (Duhamel et al. 1992; Colby and Goldberg 1999), perceptual switching during binocular rivalry (BR; Lumer et al. 1998), vestibular cortical processing (Dieterich et al. 2003), and numerical cognition (Piazza et al. 2004; Cohen Kadosh et al. 2008; Knops et al. 2009).

Specifically, numerical allocation has been shown to be linked with spatial attention mechanisms (Dehaene 1992; Fischer et al. 2003), whereby numerical magnitude is superimposed upon a left to right spatially oriented representation termed the mental number line (MNL) (Zorzi et al. 2002; Dehaene et al. 2003). This account is supported by the spatial numerical association of response code (SNARC) effect (Dehaene et al. 1993) and the observation that shifts of spatial attention follow number perception in a magnitude-dependent fashion (Fischer et al. 2003). Further, a key line of evidence for this relationship arises from observations in stroke patients with left neglect, which occurs most frequently following right fronto-parietal lesions. Patients with neglect have been shown to have relative overinhibition of the lesioned hemisphere (Corbetta and Shulman 2011) and manifest a pathological numerical bias toward larger numbers (Zorzi et al. 2002; Vuilleumier et al. 2004; Umiltà et al. 2009).

However, other research is at odds with the numerical–spatial interactions outlined above. First, the findings of Fischer and colleagues that shifts of spatial attention follow number perception in a magnitude-dependent fashion have not been replicated in more recent work (Zanolie and Pecher 2014). Further, a double dissociation between physical and number line bisection has been reported, coupled with the demonstration that the pathological number bias observed following lesions that lead to left spatial neglect are secondary to an impairment in working memory (Doricchi et al. 2005; Malhotra et al. 2005). Moreover, a recent neuroimaging study demonstrated that numerosity is topographically mapped but found no relationship to visuospatial responses (Harvey et al. 2013). Thus, experimental data to-date do not converge upon a coherent model of number–space interaction in the human brain.

Of particular relevance to the work to be presented here is the finding that patients with left spatial neglect who manifest pathological numerical biases (Zorzi et al. 2002) additionally exhibit an asymmetrical modulation of the vestibular-ocular reflex (VOR) (Doricchi et al. 2002; Ventre-Dominey et al. 2003). We have recently demonstrated that it is possible to induce handedness-related asymmetrical cortical modulation of the VOR experimentally in normal healthy subjects. This is achieved via a physiological paradigm in which subjects experience BR during concurrent vestibular stimulation that elicits left- but not right-beating vestibular nystagmus. It is currently thought that this asymmetrical modulation is associated with the relative inhibition of the left hemisphere (Arshad, Nigmatullina, and Bronstein 2013; Arshad et al. 2014; Horslen et al. 2014; Arshad et al. 2015).

Accordingly, based upon the aforementioned results from neuropsychological studies and the proposed overlapping neural networks between attentional mechanisms, vestibular function, and numerical cognition (Corbetta and Shulman 2002; Dehaene et al. 2003; Dieterich et al. 2003; Cohen Kadosh et al. 2007; Umiltà et al. 2009; van Elk and Blanke 2012), we hypothesized that numerical allocation would be subject to the same control mechanism underpinning both spatial attention and vestibular cortical processing, namely dynamic interhemispheric competition (Szczepanski and Kastner 2013; Arshad et al. 2014).

Here, we directly tested this by examining firstly whether inducing an asymmetrical modulation of the VOR following unihemispheric inhibition (confirmed using targeted noninvasive brain stimulation) could result in numerical biases toward smaller numerical magnitudes during number pair bisection (Zorzi et al. 2002). Given that previous reports conflictingly suggest that number allocation may either be intertwined or disassociated with spatial attention mechanisms (Zorzi et al. 2002; Aiello et al. 2012), we also assessed whether any numerical bias was independent from, or directly related to, a lateralized spatial attentional bias. We subsequently aimed to corroborate our findings with a computational model of numerical cognition by applying it not only to our findings, but also to those of the SNARC effect (Dehaene et al. 1993).

Taken together, this multi-method experimental approach allowed us to delineate the mechanisms underlying numerical magnitude allocation and reconcile previous experimental data to propose a unified model of numerical cognition.

### Experiment 1: Physiological Manipulation of Numerical Magnitude and Its Relationship to a Lateralized Spatial Attentional Bias

## Materials and Methods

The general experimental strategy consisted of experiencing BR during concurrent vestibular stimulation via caloric irrigation (Arshad, Nigmatullina, and Bronstein 2013; Arshad, Nigmatullina, Bhrugubanda et al. 2013).

### Vestibular Stimulation

**Figure 1.**
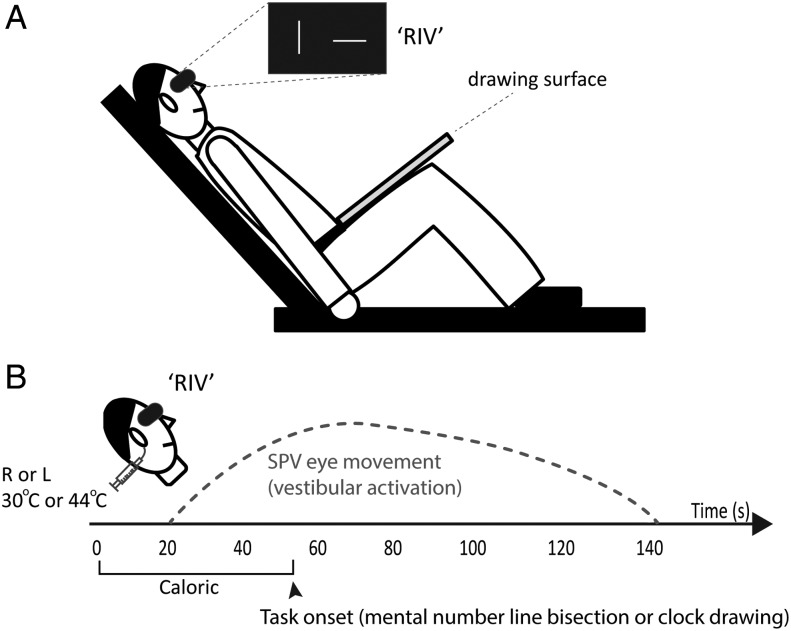
Experimental setup for number pair bisection and clock drawing (i.e., motor transformation) tasks. (*A*) Subjects lay supine with the head tilted up by 30° and with the knees flexed at 45°. The BR (“RIV”) was delivered using afterimages. A board was rested on the subject's thighs to provide writing support for the clock drawings. (*B*) Caloric irrigation (either cold 30°C or warm 44°C water irrigations) were applied to either the right (R) or left (L) ear for a duration of 40 s. Immediately at the end of the caloric irrigation, subjects performed either the mental number pair bisection task or clock drawings (Experiment 1). The vestibular activation in response to a caloric evokes nystagmus at around 20 s as represented by slow phase velocity (SPV) eye movement trace (dashed line; schematically drawn based on our normative data) (Experiment 2). In the CALORIC + RIV condition, the BR (“RIV”) was applied before the onset of the caloric and lasted for the entire duration of the task.

Participants lay supine upon a couch with the head tilted up by 30° (to obtain maximal horizontal semicircular canal activation) and both knees were flexed to 45° to provide a writing surface support for the clock drawing experiments; see below (Fig. 1*A*). The external auditory meatus was irrigated with water at either 30°C (cold) or 44°C (warm) at a rate of 500 mL/min for 40 s (CHARTR VNG: ICS medical) (Fig. 1*B*) (Cawthorne et al. 1942; Fitzgerald and Hallpike 1942).

### Visual Stimulation

As the experiment required the subjects to be in darkness to prevent vestibular suppression, BR was induced using retinal afterimages (Blake 1989) preceding the onset of vestibular activation. The rivalry device consisted of 2 LEDs illuminated at 80 cd for a duration of 40 s, positioned 42 cm directly in front of both eyes. These 2 LEDs passed a point light source through 2 striated lenses (i.e., ophthalmic Maddox rod) simultaneously, to generate a streak of light. A vertically orientated light was projected in the right eye while a horizontally orientated light was projected in the left eye (randomized between subjects) (Arshad et al. 2012). Viewing of the retinal afterimages with eyes closed resulted in rivalry lasting for 3 min, with possible percepts including vertical line (right eye image), horizontal line (left eye image), or a mixed-cross percept (i.e., image from both eyes).

### Experimental Tasks

The experimental setup remained constant for the 2 tasks that subjects performed. First, subjects performed a “mental number pair bisection task.” Two numbers were presented through a loudspeaker situated in the midline directly behind the subject. Participants were required to estimate the midpoint without calculation. Participants had to respond within 3 s to ensure no calculations were made. For each test condition, 10 trials were provided (33–87), (39–93), (44–68), (48–92), (56–92), (59–87), (61–99), (67–95), (58–124), and (58–132). Each response was noted down by the experimenter, and each of the trials was randomized between conditions (Zorzi et al. 2002). Bisection errors were calculated by subtracting the arithmetical midpoint from the reported midpoint given by the subjects, and percentage bisection errors were calculated by dividing the errors by the number interval size (Zorzi et al. 2002). Positive mean % bisection errors denoted an overestimation, whereas negative mean % bisection errors denoted an underestimation from the actual midpoint. Number pair bisection was always performed in darkness for the 3 conditions (i.e., no stimulation [BASELINE], during caloric irrigation alone [CALORIC], and during caloric irrigation combined with rivalry stimulation [CALORIC + RIV]).

The second experimental task that subjects performed was “clock drawing” to assess for any possible lateralized spatial attentional bias. Subjects were asked to draw both numerical (1–12) and alphabetical (A–L; nonnumerical control) clock faces. Clock faces were specifically chosen for their inherent right and left spatial layout, which is opposite to that found in the MNL (Aiello et al. 2012). Subjects drew clocks in both clockwise (CW) and counter-clockwise (CCW) directions, without any part of the hand touching the paper (to prevent tactile cues). Clock-face drawings were always performed in darkness for each condition (BASELINE, CALORIC, and CALORIC + RIV). Two different methods were employed to assess for distortion of clock drawings: center of Mass and inter-digit number spacing (see Supplementary Material 1).

### Subjects

A total of 40 right-handed subjects participated (Handedness score over 40 (Oldfield 1971)) (22 female, age range 18–26 years, mean age 23 years). Twenty subjects were recruited for the number pair bisection task and 20 different subjects for the clock-drawing task. In each experiment, 10 subjects participated in cold water irrigations and 10 in warm water irrigations. All subjects were naive to the purpose of study and had no history of otological, ophthalmological, psychiatric, or neurological disorders. Written informed consent was provided as approved by the local ethics research committee.

## Results

As both vestibular stimulation and switching during rivalry-viewing have been shown to shift spatial attention, which in turn can modulate numerical cognition (Rubens 1985; Fischer et al. 2003; Paffen and Van der Stigchel 2010; Ferrè et al. 2013), we first determined whether either vestibular stimulation alone, or viewing BR, induced changes in number pair bisection. No effect of rivalry-viewing or vestibular activation alone was found upon number pair bisection [rivalry versus no rivalry: *P* > 0.05, *F*_2,18_ = 0.14; vestibular stimulation versus no vestibular stimulation: *P* > 0.05, *F*_2,18_ = 0.10; left versus right caloric: *P* > 0.05, *F*_2,18_ = 0.22; repeated-measures ANOVA].

**Figure 2.**
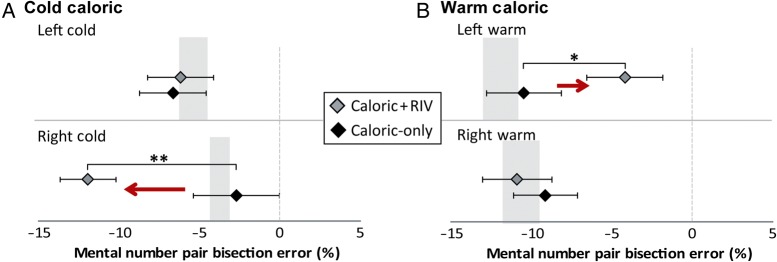
Results from mental number pair bisection experiments following physiological manipulations. We present the mean % bisection error from the midpoint of the numerical interval. (*A*) “Caloric + RIV” condition (gray diamonds) resulted in subjects significantly underestimating the midpoint (i.e., shift to the left as indicated by red arrow) compared with “Caloric-only” (black diamonds) condition following RIGHTCOLD + RIV (LOWER PANEL), but no effect was found during LEFTCOLD + RIV (upper panel). (*B*) During LEFTWARM + RIV, the subjects demonstrated a significant shift toward larger numbers (i.e., rightward shift as indicated by red arrow), suggesting overestimation of the midpoint (upper panel). No effect of RIGHTWARM + RIV was observed (lower panel). Gray-shaded area in panels indicates 95% confidence limits calculated from baseline measures (i.e., no caloric or BR stimulation). Dashed line at 0 corresponds to 0% error, i.e., accurate bisection. Data marked ** are significant at *P* < 0.01; data marked * are significant at *P* < 0.05. Error bars indicate standard errors.

We proceeded to examine whether the combination of rivalry-viewing and vestibular stimulation resulted in numerical biasing. When right ear cold irrigations, which elicit left-beating vestibular nystagmus, were combined with rivalry viewing (i.e., RIGHTCOLD + RIV), they were found to bias subjects toward smaller numbers compared with the caloric alone condition (i.e., RIGHTCOLD) (Fig. 2*A*) with a significant main effect of stimulation side [*P* < 0.003, *F*_1,9_ = 15.7, repeated-measures ANOVA] and a significant interaction between rivalry and side of stimulation [*P* < 0.005, *F*_1,9_ = 41.0]. *Post hoc* tests demonstrated a bias toward smaller numbers for the RIGHTCOLD + RIV condition (*P* < 0.001, paired *t*-test with Bonferroni correction) but no effect during left ear cold irrigations that elicit right-beating vestibular nystagmus when combined with rivalry-viewing (i.e., LEFTCOLD + RIV) (*P* = 0.71; Fig. 2*A*). Conversely, when rivalry-viewing was accompanied by left-sided warm water irrigations, which elicit left-beating vestibular nystagmus, there was a bias toward larger numbers compared with LEFTWARM caloric alone (*P* = 0.045, paired *t*-test with Bonferroni correction; Fig. 2*B*). No effect was observed during right-sided warm water irrigations (i.e., right-beating vestibular nystagmus) when combined with rivalry-viewing (RIGHTWARM + RIV) (*P* = 0.57; Fig. 2*B*).

To address whether the above reported biases were primarily due to a lateralized spatial attentional bias, subjects drew both numerical and alphabetical clock faces. If the numerical biasing we observed was directly coupled with spatial attention as per the MNL, we would expect that both numerical and alphabetical clocks would be distorted equally. Namely, we would expect a systematic leftward bias in the condition that lead to the bias toward smaller numbers (i.e., RIGHTCOLD + RIV) and a rightward bias in the condition that was associated with a bias toward larger numbers (i.e., LEFTWARM + RIV).

**Figure 3.**
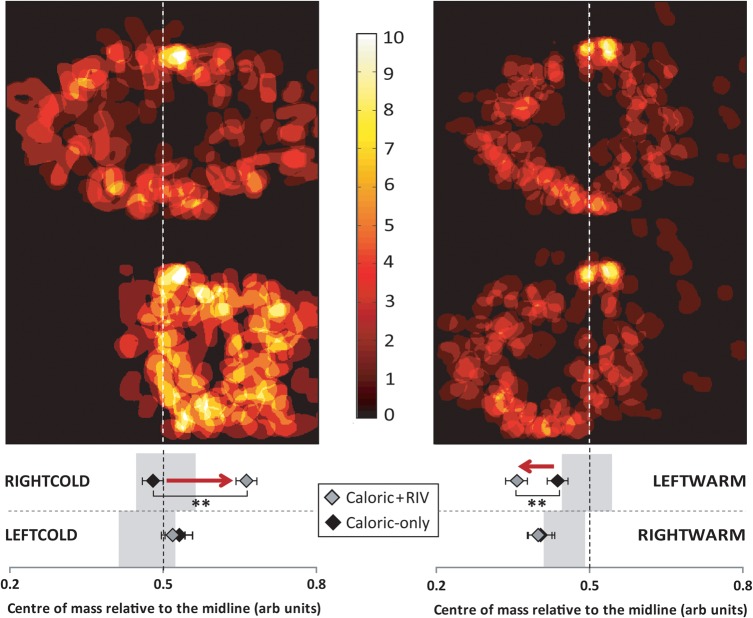
Heat maps (upper panel) illustrate numerical clock-drawing performance in caloric-only conditions (RIGHTCOLD on top left; LEFTWARM on top right) with corresponding ‘+RIV’ conditions below. Center of mass results are displayed in the lower panel: (*A*) (left panel) Following RIGHTCOLD + RIV when subjects were asked to draw the clocks clockwise (CW), a significant shift to the right (indicated by red arrow) is seen in the “Caloric + RIV” condition (gray diamonds) compared with “caloric alone” (black diamond) condition. (*B*) (right panel) Following LEFTWARM + RIV when subjects were asked to draw the clock anticlockwise (ACW), a significant shift to the left (indicated by red arrow) was observed in “Caloric + RIV” (grey diamonds) compared with “caloric alone” (black diamond) condition. Gray-shaded area in lower panels indicates 95% confidence limits calculated from baseline measures (i.e., no caloric or BR stimulation). Dashed line at 0.5 indicates the midline of a perfectly symmetrical clock. Data marked ** are significant at *P* < 0.01. Error bars indicate standard errors.

Figure 3 illustrates that numerical clocks drawn clockwise in the RIGHTCOLD + RIV condition were laterally displaced to the right-hand side of space (Fig. 3 and see Supplementary Fig. 2 upper panel). A 2 × 3 ANOVA examining displacement [factors: side (left, right), and condition (BASELINE, CALORIC, CALORIC + RIV)] showed no main effect for side; however, there was a significant effect for condition (*P* < 0.001, *F*_2,18_ = 12.3) and also an interaction between side and condition (*P* < 0.008, *F*_2,18_ = 6.4). *Post hoc* tests revealed no effects for the RIGHTCOLD-only condition but a significant effect for RIGHTCOLD + RIV versus baseline (*P* < 0.008) and CALORIC + RIV versus caloric alone (*P* = 0.005, paired *t*-test Bonferroni adjusted; caloric-only versus baseline: *P* = 0.73; Fig. 3). Conversely, numerical clocks for LEFTWARM + RIV drawn anticlockwise were laterally displaced leftwards (Fig. 3 and see Supplementary Fig. 2 lower panel). As in the previous analysis, ANOVA revealed a main effect of condition (*P* < 0.0001, *F*_2,18_ = 21.7) and a significant interaction between side and condition (*P* < 0.003, *F*_2,18_ = 8.4). *Post hoc* tests demonstrated no effect for the LEFTWARM-only condition but a significant effect between LEFTWARM + RIV versus baseline (*P* < 0.001), CALORIC + RIV versus caloric alone (*P* < 0.007) (see Fig. 3).

Notably, neither RIGHTWARM + RIV nor LEFTCOLD + RIV conditions distorted numerical clock drawings. Critically, drawing alphabet clocks clockwise during cold caloric irrigation showed no significant effect of the side of the irrigation (*F*_2,18_ = 0.08, *P* > 0.05) or condition (BASELINE, CALORIC, CALORIC + RIV; *F*_2,18_ = 1.29, *P* > 0.05, 2 × 3 ANOVA). Similarly, no significant effects were found during warm caloric irrigation (side: *F*_2,18_ = 0.001, *P* > 0.05; conditions: *F*_2,18_ = 5.1, *P* > 0.05). For anticlockwise alphabet clock drawings, there were also no significant effects found for either cold (side: *F*_2,18_ = 0.031, *P* > 0.05; conditions: *F*_2,18_ = 1.1, *P* > 0.05) or warm caloric irrigations (side: *F*_2,18_ = 0.34, *P* > 0.05; conditions: *F*_2,18_ = 2.0, *P* > 0.05).

Our prediction in Experiment 1 was that both RIGHTCOLD + RIV and LEFTWARM + RIV conditions would modulate numerical cognition in the same direction, as we predicted that in both of these conditions, following interhemispheric conflict, one would expect inhibition of the same hemisphere (i.e., left hemisphere) (Arshad et al. 2014, 2015). However, we serendipitously observed a differential modulation of numerical allocation, as RIGHTCOLD + RIV biased subjects toward smaller numbers, whereas LEFTWARM + RIV biased subjects toward larger numbers. Moreover, clock-face drawings were also distorted and laterally displaced in opposing directions by the 2 conditions.

**Figure 4.**
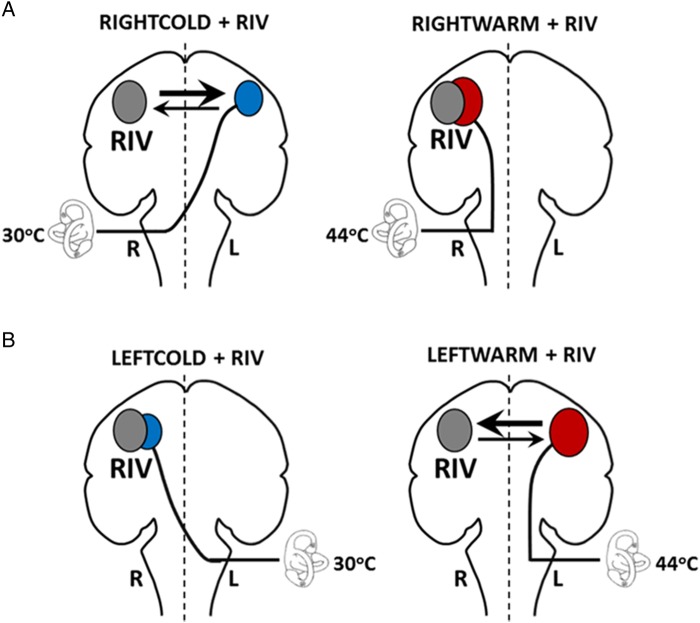
Schematic model illustrating proposed hemispheric activation in the Caloric + RIV condition. The perceptual switching in BR (RIV) is proposed to activate the right hemisphere (gray circle). Hemispheric activations following caloric stimulation are shown by the red circle following warm irrigations or by blue circles following cold irrigations. The labyrinth represents the side of the caloric irrigation. The size of the circles illustrates the relative degree of the activation. (*A*) In the RIGHTCOLD + RIV condition, the hemispheres are in conflict; however, the right hemisphere exerts a predominant effect (as shown by the relative thickness of the arrows). The interhemispheric conflict is not present during the RIGHTWARM + RIV condition as the right hemisphere is preferentially activated by both the visual and vestibular stimuli. (*B*) Similarly, no conflict is present in LEFTCOLD + RIV condition, whereas during the LEFTWARM + RIV condition conflict presents, but critically here the left hemisphere exerts a greater influence during the interhemispheric conflict.

These results raise the critical question as to why RIGHTCOLD + RIV and LEFTWARM + RIV led to opposing effects. The underlying principle of this technique is that during concurrent visuo-vestibular stimulation it is possible to selectively induce interhemispheric conflict solely by altering the vestibular stimulus (Arshad, Nigmatullina, Bhrugubanda et al. 2013). Previous studies implementing either functional imaging approaches or behavioral neuro-modulatory techniques have shown that neural activity associated with perceptual switching during BR is tightly linked to a fronto-parietal network, predominantly in the right hemisphere (Lumer et al. 1998; Corbetta and Shulman 2002; Carmel et al. 2010; Zaretskaya et al. 2010; Knapen et al. 2011). Moreover, we have shown both previously and herein (see Supplementary Material 3) that identical effects can be obtained if other visuospatial paradigms that call upon the right hemisphere are combined with vestibular stimulation (Arshad, Nigmatullina, Bhrugubanda et al. 2013; Arshad, Nigmatullina, and Bronstein 2013). Interhemispheric conflict is induced when the vestibular component is predominantly processed in the left hemisphere (i.e., a right-sided cold [RIGHTCOLD] or left-sided warm [LEFTWARM] caloric irrigation) (Suzuki et al. 2001; Dieterich et al. 2003; Lopez et al. 2012; Zu Eulenburg et al. 2012). In the aforementioned scenarios, an asymmetrical VOR is induced (Arshad, Nigmatullina, Bhrugubanda et al. 2013) but when vestibular stimulation induces predominantly right hemisphere activation, (i.e., left-sided cold [LEFTCOLD] or right-sided [RIGHTWARM] irrigations) (Suzuki et al. 2001; Dieterich et al. 2003; Lopez et al. 2012; Zu Eulenburg et al. 2012), there is no interhemispheric conflict, as both the vestibular and visual components preferentially activate the same hemisphere. Accordingly, we hypothesized that these opposing effects were attributable to the comparative difference in the degree of left hemisphere vestibular activation during RIGHTCOLD and LEFTWARM irrigations, respectively (Fig. 4) (Akbarian et al. 1988, 1992, 1993, 1994). This relative difference would then, via an “all-or-nothing” effect, determine which hemisphere is inhibited by concurrent visuo-vestibular stimulation. We directly tested this hypothesis in Experiment 2.

### Experiment 2: Using Transcranial Direct Current Stimulation to Probe the Neural Correlates of the Asymmetrical VOR Modulation

To test the above hypothesis, we applied unipolar transcranial direct current stimulation (tDCS) over the frontal eye fields to either augment or attenuate the VOR asymmetries following CALORIC + RIV stimulation. This region was chosen as the stimulation site as it has previously been demonstrated as a critical node in fronto-parietal networks underlying numerical processing, vestibular processing and for the control of spatial attention (Husain and Kennard 1996; Jahanshahi et al. 2000; Kluge et al. 2000; Fasold et al. 2002; Nieder and Miller 2004; Corbetta et al. 2005; Nieder 2005; Nieder and Dehaene 2009). Crucially, unlike parietal tDCS alone, direct stimulation of the frontal eye fields does not lead to modulation of the VOR (Arshad et al. 2014, 2015). Thus, any effect on the VOR would be secondary to modulation of interhemispheric interactions rather than any direct influence on vestibular processing. During this experiment, participants were exposed to identical stimulation conditions to those employed in Experiment 1, with simultaneous eye movement recording. (Arshad, Nigmatullina, Bhrugubanda et al. 2013; Arshad, Nigmatullina, and Bronstein 2013). We predicted that if the right hemisphere was primarily involved in mediating the VOR modulation during the RIGHTCOLD + RIV condition, then unipolar anodal (i.e., excitatory) stimulation of the right hemisphere and unipolar cathodal stimulation (i.e., inhibition) of the left hemisphere would augment the VOR asymmetries, whereas unipolar left hemisphere anodal and unipolar right hemisphere cathodal stimulation would lead to attenuation. If the left hemisphere were mediating the effects during the LEFTWARM + RIV condition, we would expect the opposite effects: attenuation of VOR asymmetries with unipolar anodal stimulation of the right hemisphere and unipolar cathodal stimulation of the left hemisphere and augmentation with unipolar left hemisphere anodal and unipolar right hemisphere cathodal stimulation.

## Materials and Methods

### Eye Movement Recording

LEFTCOLD and RIGHTWARM irrigations elicit right-beating nystagmus, whereas LEFTWARM and RIGHTCOLD elicit left-beating nystagmus (Cawthorne et al. 1942; Fitzgerald and Hallpike 1942). The oculomotor response following vestibular stimulation was tracked using a head mounted infra-red binocular video-oculography (VOG) system. An automated computerized program (CHARTR VNG; ICS medical) removed the fast phases from the nystagmus waveform, allowing us to plot the velocity of each nystagmic slow phase over 120 s (Fig. 1*B*). Response intensity was determined by obtaining the mean peak slow phase eye velocity (Barnes 1995).

### Transcranial Direct Current Stimulation

A battery-driven stimulator (neuroConn GMBH, Ilmenau, Germany) was used to apply stimulation. The current had a ramp up time of 10 s at which point a constant current of 1.5 mA was applied for a duration of 15 min. At the end of the stimulation, the current was ramped down in a 10 s fade out period. The uni-hemispheric tDCS montage chosen was the same as that used in a previous study that targeted the frontal eye fields that lie within the dorsolateral pre-frontal cortex (dLPFC) (Kanai et al. 2012). Electrode positions were defined using 10–20 international EEG electrode placement coordinates. That is, for either ANODAL or CATHODAL stimulation of the right hemisphere, the electrode was placed over F4 (10–20 EEG coordinate), while for either ANODAL or CATHODAL stimulation of the left hemisphere the electrode was placed over F3 (10–20 EEG coordinate). The reference electrode was always placed over the ipsilateral shoulder (deltoid muscle) (Kanai et al. 2012).

### Experimental Protocol

First, we confirmed that when BR is combined with left-beating vestibular nystagmus, it induces an asymmetrical VOR (Arshad, Nigmatullina, and Bronstein 2013). Two groups of 10 right-handed subjects (Handedness score over 40) were recruited; Group 1: cold water irrigations (5 males; age range 20–26, mean age 21.9); Group 2: warm water irrigations (6 females; age range 20–24, mean age 21.3). Both groups underwent 4 conditions in total: cold (Group 1) or warm (Group 2) CALORIC alone on the right, cold (Group 1) or warm (Group 2) CALORIC alone on the left, cold (Group 1) or warm (Group 2) RIGHTCALORIC + RIV and cold (Group 1) or warm (Group 2) LEFTCALORIC + RIV. In each condition, we established the peak slow phase eye velocity (SPV). We compared the peak SPV for the CALORIC + RIV condition with the corresponding CALORIC alone condition (Arshad et al. 2014).

In the second part of the experiment, we modulated cortical excitability using unipolar frontal tDCS in 4 separate randomized sessions, with each session separated by 4 days to avoid carryover effects. For each group, we assessed VOR asymmetries following both right hemisphere anodal or cathodal stimulation, and both left hemisphere anodal or cathodal stimulation.

## Results

**Figure 5.**
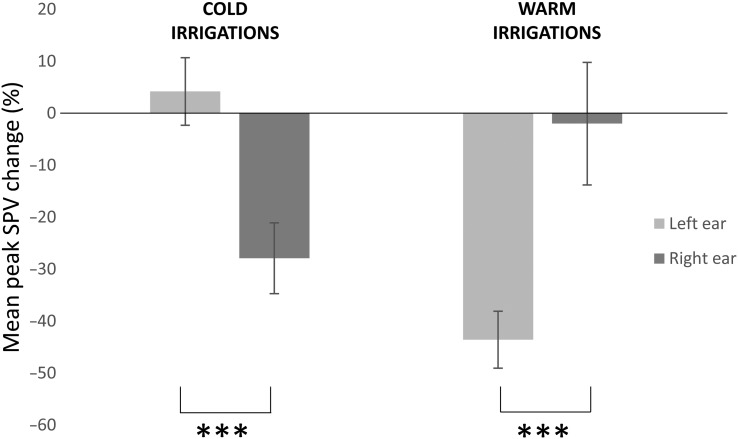
Asymmetrical modulation of the VOR during combined caloric irrigation and rivalry-viewing. On the *y*-axis, we represent the mean % change in peak SPV when comparing the CALORIC alone condition with the corresponding CALORIC + RIV condition. On the *x*-axis, we have represented the different conditions, namely cold or warm water irrigations of either the right (dark gray bar) or left (light gray bar) ear. Note that we observe a marked suppression of the VOR for the following conditions, RIGHTCOLD + RIV compared with RIGHTCOLD irrigations alone and LEFTWARM + RIV compare with LEFTWARM irrigations alone. No suppression of the VOR was observed when comparing LEFTCOLD + RIV with LEFTCOLD irrigations alone or RIGHTWARM + RIV to RIGHTWARM irrigations alone. Data marked *** are significant at *P* < 0.001. Error bars indicate standard error.

For Group 1, a 2 × 2 repeated-measures ANOVA with BR (2 levels: BR, no BR) and laterality of caloric (2 levels; left ear, right ear) indicated a significant main effect of BR (*F*_1,9_ = 34.5, *P* < 0.0001), no main effect of laterality (*F*_1,9_ = 1.2, *P* > 0.05), and a significant interaction between laterality × rivalry (*F*_1,9_ = 7.8, *P* = 0.021) (Fig. 5). *Post hoc* paired *t*-tests (Bonferroni corrected) revealed a significant difference between RIGHTCOLD alone and RIGHTCOLD + RIV (*P* < 0.0001; paired *t*-test). No effect was observed for LEFTCOLD irrigations (*P* > 0.05; paired *t*-test). In Group 2, a separate 2 × 2 repeated-measures ANOVA with BR (2 levels) and laterality of caloric (2 levels) indicated a significant main effect of rivalry (*F*_1,9_ = 8.1, *P* = 0.019), no significant main effect of laterality (*F*_1,9_ = 1.0, *P* > 0.05), and a significant interaction between laterality × rivalry (*F*_1,9_ = 8.7, *P* = 0.016) (Fig. 5). *Post hoc* paired *t*-tests (Bonferroni corrected) revealed a significant difference between LEFTWARM alone and LEFTWARM + RIV (*P* < 0.0001; paired *t*-test). No effect was observed for RIGHTWARM irrigations (*P* > 0.05; paired *t*-test).

**Figure 6.**
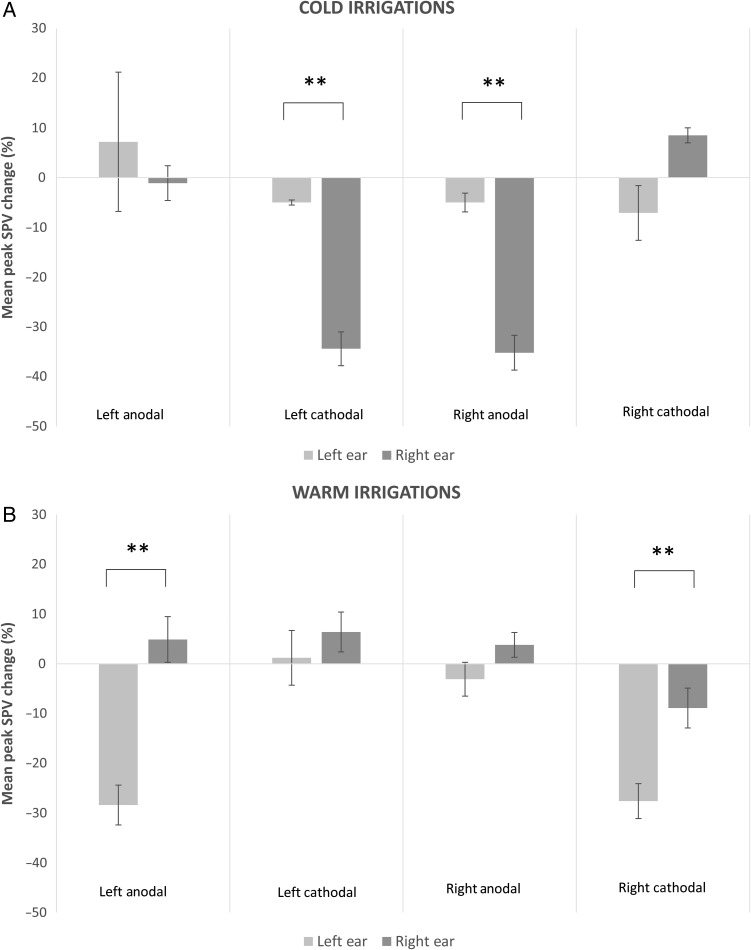
Probing the neural correlates of the asymmetrical VOR modulation following combined CALORIC + RIV stimulation using tDCS. (*A*) Top panel represents the results from the cold water irrigations (i.e., Group 1). On the *y*-axis, we represent the mean % change in peak SPV when comparing the CALORIC alone condition with the corresponding CALORIC + RIV condition. On the *x*-axis, we have represented the different conditions of either the right (dark gray bar) or left (light gray bar) ear cold water irrigations following unipolar left anodal, left cathodal, right anodal, or right cathodal stimulation. Note that for RIGHTCOLD + RIV, we only observed asymmetries of the VOR following unipolar right hemisphere anodal stimulation and unipolar left hemisphere cathodal stimulation. Note that the asymmetries in the VOR during RIGHTCOLD + RIV were attenuated following either unipolar anodal stimulation of the left hemisphere or unipolar cathodal stimulation of the right hemisphere. (*B*) Lower panel represents the results from the warm water irrigations (i.e., Group 2). Again on the *y*-axis, we represent the mean % change in peak SPV when comparing the CALORIC alone condition with the corresponding CALORIC + RIV condition. On the *x*-axis, we have represented the different conditions of either the right (dark gray bar) or left (light gray bar) ear warm water irrigations following unipolar left anodal, left cathodal, right anodal, or right cathodal stimulation. Note that for LEFTWARM + RIV, we only observed asymmetries of the VOR following either unipolar left hemisphere anodal stimulation or unipolar right hemisphere cathodal stimulation. Note that the asymmetries in the VOR during LEFTWARM + RIV were attenuated following either unipolar anodal stimulation of the right hemisphere or unipolar cathodal stimulation of the left hemisphere. Data marked ** are significant at *P* < 0.01. Error bars indicate standard errors.

For the second part of the experiment, a 2 × 2 × 2 × 2 repeated-measures ANOVA for Group 1 was employed, with factors SIDE (2 levels; right ear or left ear), RIVALRY (2 levels; BR or no BR), STIMULATION TYPE (2 levels; cathodal or anodal) and STIMULATION SIDE (2 levels; left hemisphere or right hemisphere). This revealed a significant main effect for caloric side (*F*_1,9_ = 129, *P* < 0.001), significant main effect of BR (*F*_1,9_ = 20.70, *P* < 0.001), no main effect for stimulation type (*F*_1,9_ = 1.7, *P* > 0.05), and a significant main effect for stimulation side (*F*_1,9_ = 4.97, *P* = 0.04). There was a significant 4-way interaction between side × rivalry × stimulation type × stimulation side (*F*_1,9_ = 89.19, *P* < 0.0001) (Fig. 6*A*). *Post hoc* paired *t*-tests (Bonferroni corrected) revealed that in the RIGHTCOLD + RIV condition, application of either right hemisphere anodal stimulation or left hemisphere cathodal stimulation induced asymmetrical modulations of the VOR (*P* < 0.001; paired *t*-test). No asymmetries of the VOR were induced for the RIGHTCOLD + RIV condition following either right hemisphere cathodal or left hemisphere anodal stimulation (*P* > 0.05; paired *t*-test). Further, no effect for LEFTCOLD irrigations was observed in any of the tDCS conditions (*P* > 0.05; paired *t*-test) (Fig. 6*A*).

As in the previous analysis, a 2 × 2 × 2 × 2 repeated-measures ANOVA for Group 2 revealed a significant main effect for caloric side (*F*_1,9_ = 137.4, *P* < 0.001), significant main effect of BR (*F*_1,9_ = 24.6, *P* < 0.001), no main effect for stimulation type (*F*_1,9_ = 0.835, *P* > 0.05), and a significant main effect for stimulation side (*F*_1,9_ = 3.84, *P* = 0.047). There was a significant 4-way interaction between side × rivalry × stimulation type × stimulation side (*F*_1,9_ = 77.17, *P* < 0.0001) (Fig. 6*B*). *Post hoc* paired *t*-tests (Bonferroni corrected) revealed that in the LEFTWARM + RIV condition, application of either right hemisphere cathodal stimulation or left hemisphere anodal stimulation induced asymmetrical modulation of the VOR (*P* < 0.001; paired *t*-test). No asymmetries of the VOR were induced for the LEFTWARM + RIV condition following either right hemisphere anodal or left hemisphere cathodal stimulation (*P* > 0.05; paired *t*-test). Further, no effect for RIGHTWARM irrigations were observed in any of the tDCS conditions (*P* > 0.05; paired *t*-test) (Fig. 6*B*).

Taken together, these results provide strong evidence that in the 2 conditions that induce interhemispheric conflict and subsequent asymmetrical modulation of the VOR, namely RIGHTCOLD + RIV and LEFTWARM + RIV, there is selective inhibition of the left and right hemispheres, respectively. That is, during RIGHTCOLD + RIV, the asymmetrical modulation of the VOR is mediated by the right hemisphere, as anodal stimulation of the right hemisphere and cathodal stimulation of the left hemisphere augment the VOR asymmetries, whereas left hemisphere anodal and right hemisphere cathodal stimulation attenuate the VOR asymmetries (Figs 4 and 6). Conversely, during LEFTWARM + RIV, the asymmetrical modulation of the VOR is mediated by the left hemisphere, as left hemisphere anodal stimulation and cathodal stimulation of the right hemisphere augment the VOR asymmetries, whereas left hemisphere cathodal or right hemisphere anodal stimulation attenuates the VOR asymmetries (Figs 4 and 6). Hence, these findings are in keeping with the results of Experiment 1 and provide an explanation for the opposing effects upon number allocation of right-sided cold water irrigation and left-sided warm water irrigation, when combined with binocular rivalry-viewing. Accordingly, we proceeded to examine the precise relationship between induced VOR asymmetries, uni-hemispheric inhibition, and numerical magnitude allocation in Experiment 3.

### Experiment 3: Relationship Between Uni-Hemispheric Inhibition, Induced VOR Asymmetries, and Numerical Magnitude Allocation

#### Experiment 3a: Relationship Between VOR Asymmetries and Numerical Biases

In the above group of subjects (i.e., those that participated in Experiment 2), we proceeded to examine the relationship between the degree of VOR suppression induced by either the RIGHTCOLD + RIV or LEFTWARM + RIV conditions, respectively, upon both 1) each individuals mean number pair bisection error (%) and 2) the size of the lateral shift induced in the center of mass for the numerical clock drawings.

**Figure 7.**
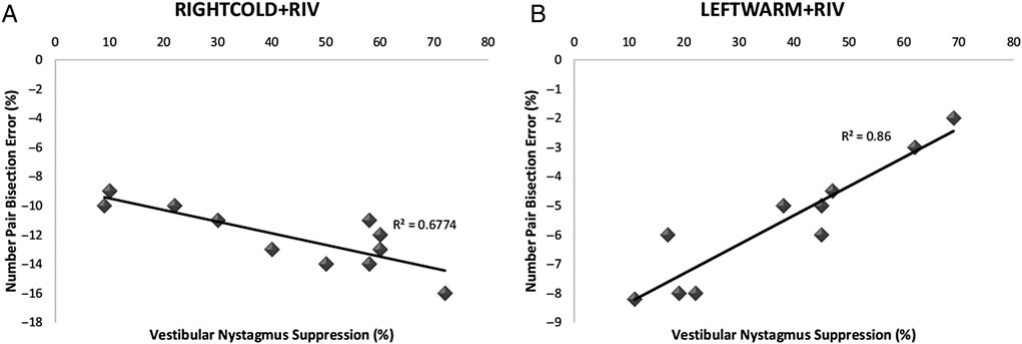
Relationship between numerical perceptual biases and degree of VOR suppression. (*A*) On the *x*-axis, we present the degree of vestibular nystagmus suppression (i.e., % change in SPV) between right cold caloric alone and RIGHTCOLD + RIV. On the *y*-axis, we represent the number pair bisection error (%). We observed a significant negative correlation between the number pair bisection error (i.e., bias toward smaller numbers) and the degree of vestibular nystagmus suppression. That is, those individuals who exhibited a larger bias toward smaller numbers during RIGHTCOLD + RIV also demonstrated a larger degree of vestibular nystagmus suppression. (*B*) On the *x*-axis, we present the degree of vestibular nystagmus suppression (i.e., % change in SPV) between left warm caloric alone and LEFTWARM + RIV. On the *y*-axis, we represent the number pair bisection error (%).We observed a significant positive correlation between the number pair bisection error (i.e., bias toward larger numbers) and the degree of vestibular nystagmus suppression. That is, those individuals who exhibited a more pronounced bias toward larger numbers demonstrated greater vestibular nystagmus suppression.

As shown in Figure 7, we observed a significant negative correlation between number pair bisection error (%) and the degree of vestibular nystagmus suppression following RIGHTCOLD + RIV (*R*^2^ 0.6774, *P* < 0.01 Pearson's correlation), whereas following LEFTWARM + RIV, we observed a significant positive correlation (*R*^2^ 0.86, *P* < 0.01 Pearson's correlation). That is, following RIGHTCOLD + RIV, the individuals who exhibit greater degree of vestibular nystagmus suppression demonstrated a more pronounced numerical bias toward smaller numbers. Conversely, following LEFTWARM + RIV, individuals who exhibited greater vestibular nystagmus suppression demonstrated a more pronounced numerical bias toward larger numbers (Fig. 7). These finding are in line with our recent observations, which demonstrate that interhemispheric asymmetries as reflected by vestibular nystagmus suppression can directly predict individual differences in line bisection error (i.e., pseudoneglect) (Arshad et al. Forthcoming).

**Figure 8.**
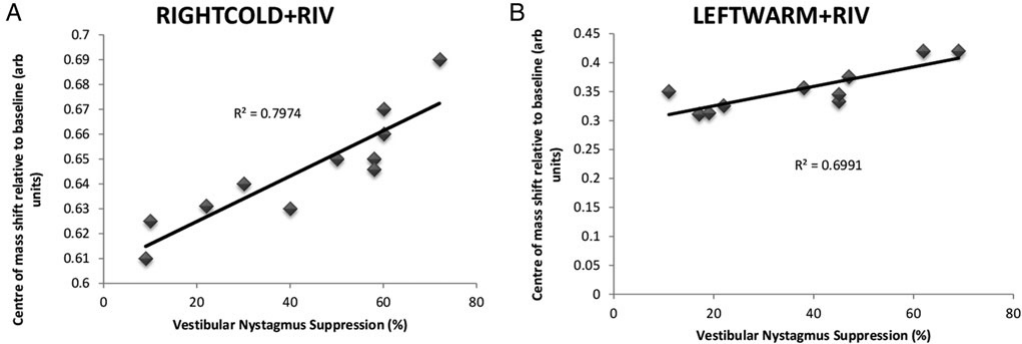
Relationship between lateral shifts observed during numerical clock drawings and degree of VOR suppression. On the *x*-axis, we represent the degree of vestibular nystagmus suppression and on the *y*-axis we represent the relative shift in the center of mass (arbitrary units). (*A*) For RIGHTCOLD + RIV, we observed a positive correlation, in that those individual who exhibited greater VOR asymmetries had larger shifts in the center of mass to the right. (*B*) For LEFTWARM + RIV, we also observed a positive correlation, in that those individual who exhibited greater VOR asymmetries had larger shifts in the center of mass to the left.

With respect to the numerical clock drawings, the larger the rightward lateral shift during RIGHTCOLD + RIV, the greater the vestibular nystagmus suppression (*R*^2^ 0.7974, *P* < 0.01 Pearson's correlation). During LEFTWARM + RIV, a greater degree of vestibular nystagmus suppression was associated with a more pronounced leftward lateral shift (*R*^2^ 0.6991, *P* < 0.01 Pearson's correlation) (Fig. 8).

The above data directly demonstrate that a correlative relationship exists between the numerical effects observed in Experiment 1 and the degree of eye movement suppression induced by the CALORIC + RIV stimulation, as tested in part 1 of Experiment 2 (i.e., Fig. 5). Having established this relationship, we proceeded to apply frontal tDCS to modulate the VOR asymmetries, as per the stimulation paradigm in the second part of Experiment 2, to ascertain its impact upon numerical magnitude perception.

#### Experiment 3b: Using tDCS to Probe the Neural Correlates of Numerical Magnitude Allocation

To specifically ascertain the neuro-anatomical correlates of numerical magnitude allocation, following CALORIC + RIV stimulation, we applied unipolar frontal tDCS. During this experiment, participants were exposed to an identical stimulation paradigm and experimental task (i.e., number pair bisection task) to that employed in Experiment 1.

We predicted that, if relative unihemispheric inhibition following hemispheric conflict was responsible for the numerical biases, then tDCS, by increasing or decreasing the degree of resultant unihemispheric inhibition, would augment or reverse the numerical bias in the conflict conditions (i.e., RIGHTCOLD + RIV and LEFTWARM + RIV) with a directional specificity. Moreover, we predicted that simply modulating cortical excitability via application of tDCS either alone or in the no-conflict conditions (i.e., LEFTCOLD + RIV and RIGHTWARM + RIV) would have no effect upon numerical magnitude allocation.

Application of unipolar tDCS alone had no effect upon number pair bisection error. A 2 × 2 repeated-measures ANOVA revealed no main effect for either side of stimulation (*F*_9,1_ = 0.110, *P* > 0.05) nor type of stimulation (*F*_9,1_ 2.276, *P* > 0.05) (see Supplementary data and Fig. 4). Subsequently, to assess the effects of tDCS upon number pair bisection during the CALORIC + RIV conditions, we recruited 2 groups of 10 right-handed subjects (Handedness score over 40). Group 1 participated in cold water irrigations (6 males; age range 19–26, mean age 22.7), whereas Group 2 participated in warm water irrigations (4 females; age range 20–28, mean age 23.3). For each group, we compared the number pair bisection error during CALORIC + RIV stimulation relative to the corresponding caloric alone condition, both before and after tDCS. Cortical excitability was modulated using unipolar frontal tDCS as performed in Experiment 2. This constituted 4 separate randomized sessions (i.e., right hemisphere anodal or cathodal stimulation, and left hemisphere anodal or cathodal stimulation), with each session separated by 4 days to avoid any potential carryover effects.

**Figure 9.**
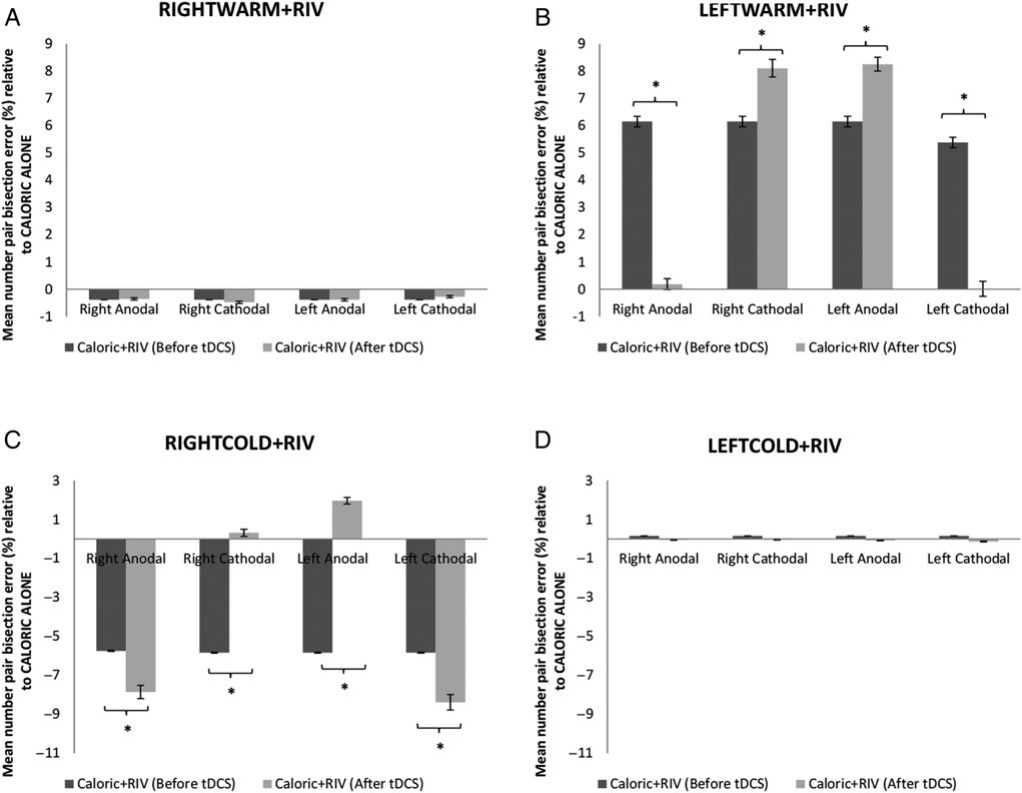
Summary of the results for the effects of frontal tDCS upon numerical magnitude allocation. On the *y*-axis, we represent the mean change in bisection error (%) when comparing caloric alone with the corresponding CALORIC + RIV condition, either before (dark gray bars) or after (light gray bars) application of tDCS. On the *x*-axis, we represent the 4 different tDCS stimulation paradigms implemented. (*A*) No effect of tDCS upon number pair bisection was observed in any of the 4 stimulation conditions during RIGHTWARM + RIV. (*B*) For LEFTWARM + RIV, there was a bias toward larger numbers before application of tDCS, which was abolished following unipolar right anodal and left cathodal stimulation. Notably, this bias toward larger numbers was augmented following either unipolar left anodal or right cathodal stimulation. (*C*) During RIGHTCOLD + RIV, there was a bias toward smaller numbers before tDCS; however, this bias was abolished following either unipolar left anodal stimulation or right cathodal stimulation and augmented following either unipolar right anodal or left cathodal stimulation, respectively. (*D*) No effect of tDCS was observed upon number pair bisection in any of the 4 stimulation conditions during LEFTCOLD + RIV. Data marked * significant at *P* < 0.001. Error bars indicate standard errors.

For Group 1, a 2 × 2 × 2 × 2 repeated-measures ANOVA was employed, with factors TYPE of stimulation (2 levels; anodal or cathodal), SIDE of stimulation (2 levels; right or left), IRRIGATION side (2 levels; right or left), and TIME (2 levels; number pair bisection error either before or after tDCS). This revealed a significant main effect for TIME (*F*_1,9_ = 21.4, *P* < 0.001), a significant main effect for IRRIGATION side (*F*_1,9_ = 47.49, *P* < 0.000), a significant main effect for SIDE of stimulation (*F*_1,9_ = 2.41, *P* < 0.05), but no main effect for TYPE of stimulation (*F*_1,9_ = 0.018, *P* > 0.05). There was a significant 4-way interaction between type × side × irrigation × time (*F*_1,9_ = 59.149, *P* < 0.0001) (Fig. 9*C*). *Post hoc* paired *t*-tests (Bonferroni corrected) revealed that in the RIGHTCOLD + RIV condition, application of either right hemisphere anodal stimulation or left hemisphere cathodal stimulation augmented the numerical biases toward smaller numbers (*P* < 0.001; paired *t*-test). Further, during RIGHTCOLD + RIV, the numerical biasing toward smaller numbers was abolished following either right hemisphere cathodal or left hemisphere anodal stimulation (*P* > 0.05 paired *t*-test) (Fig. 9*C*). No effects upon number pair bisection error were observed for LEFTCOLD irrigations in any of the tDCS conditions (*P* > 0.05; paired *t*-test) (Fig. 9*D*).

For Group 2, as in the previous analysis, a 2 × 2 × 2 × 2 repeated-measures ANOVA revealed a significant main effect for TIME (*F*_1,9_ = 30.49, *P* < 0.0001), a significant main effect for IRRIGATION side (*F*_1,9_ = 90.7, *P* < 0.0001), a significant main effect for SIDE of stimulation (*F*_1,9_ = 0.2193, *P* < 0.05), but no main effect for TYPE of stimulation (*F*_1,9_ = 0.153, *P* > 0.05). There was a significant 4-way interaction between type × side × irrigation × time (*F*_1,9_ = 287.53, *P* < 0.0001) (Fig. 9*B*). *Post hoc* paired *t*-tests (Bonferroni corrected) revealed that in the LEFTWARM + RIV condition, application of either left hemisphere anodal stimulation or right hemisphere cathodal stimulation augmented the numerical biases toward larger numbers (*P* < 0.001; paired *t*-test). Further, during LEFTWARM + RIV, the numerical biasing toward larger numbers was abolished following either left hemisphere cathodal or right hemisphere anodal stimulation (*P* > 0.05; paired *t*-test). No effects upon number pair bisection error were observed for RIGHTWARM irrigations in any of the tDCS conditions (*P* > 0.05; paired *t*-test) (Fig. 9*A*).

Taken together, these data provide a direct demonstration that RIGHTCOLD + RIV results in left hemisphere inhibition and numerical biases toward smaller numbers, whereas LEFTWARM + RIV results in right hemisphere inhibition which biases judgements toward larger numbers.

### Experiment 4: Computational Model of Numerical Allocation

Following on from the findings that left hemisphere inhibition was associated with numerical biasing toward smaller numbers and right hemisphere inhibition with bias toward larger numbers, we sought a mathematical model that could predict the biases observed. We implement *x* to denote the percentage error in midpoint bisection and *p(x)* to denote the probability of this error. The distribution *p(x)* is affected only by the hemispheric conflict conditions (i.e., RIGHTCOLD + RIV and LEFTWARM + RIV). Total stimulation of the right hemisphere is denoted by *r* and total stimulation of the left hemisphere by *l*. The probability of making an error *p(x)* in the bisection task depends on both *r* and *l* (i.e., *p(x)* = *p(x;l,r)*). We implement a statistical mechanical model, such that for *p(x;l,r)* we can represent it as a Boltzmann weight, whereby *β* is the parameter specifying the width of the probability distribution and *E(x;l,r)* is a function (i.e., energy). The denominator applied in equation [1] is a normalization factor.

(1) $$p(x;l,r)=\frac{\exp(-E(x;l,r)\beta )}{\int_{-\infty }^{\infty }\exp(-E(x;l,r)\beta )\text{d}x}.$$

The choice of the function *E*(*x;l,r*) completes the construction of the model as follows:

(2) $$E(x;l,r)=(1-lr)x^{2}+(-l^{2}r+lr^{2})x+(1+lr)x^{4}.$$

**Figure 10.**
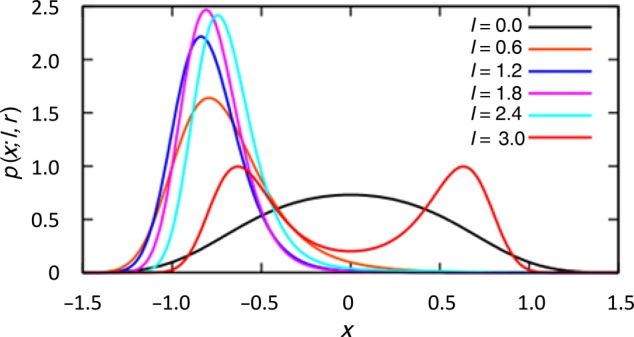
Computational modelling. The figure illustrates the probability distribution *p(x; l,r)* that occurs for several different values of *l* where the following parameters were implemented in the model *r* = 3.0 and *β* = 1.

Both equations [1] and [2] can completely define the model and allow the calculation of various bisection errors based upon the strength of right and left hemisphere activation, respectively. Each term in equation [2] has a physical meaning so that the first term is quadratic in *x* and when either *l* or *r* or both are equal to zero, it simply penalizes any deviations from the optimal value *x* = 0 as found during no hemispheric conflict conditions (i.e., LEFTCOLD + RIV or RIGHTWARM + RIV). In hemispheric conflict conditions (i.e., RIGHTCOLD + RIV or LEFTWARM + RIV), both *l* and *r* are concurrently nonzero leading to the bisection error shifts. During conflict, having *x* = 0 is no longer the optimum value and the most likely bisection error is shifted toward either smaller or larger numbers. Due to the second term in equation [2], the shift observed is asymmetric. That is, in conflict situations only the relatively greater activation of the right hemisphere results in a bisection error shift toward smaller numbers (negative direction), whereas left hemisphere activation following conflict shifts the error in the positive direction (i.e., larger numbers). The last term in equation [2] is implemented to ensure that very large deviations of *x* from 0 are unfavorable, even in the presence of large interhemispheric conflict (i.e., ceiling effect). Figure 10 illustrates several calculated probability distributions that correspond to a fixed value of *r*, but different values of *l* and hence varying degree of the interhemispheric competition. When *r* and *l* are equal, either hemisphere may be preferentially activated. Accordingly, the subject is equally likely to make errors in either the positive or negative direction. To confirm that the model generalized to other experimental findings, we verified it by applying it to the most influential account of lateralized processing and numerical cognition: the SNARC effect (Dehaene et al. 1993) (see Supplementary Material 6).

## Discussion

Using an innovative multi-method approach to induce dynamic interhemispheric competition in neurologically intact individuals, we sought to investigate how the brain controls numerical magnitude. Implementation of this methodology allowed us to avoid the associated confounds of previous studies that have probed numerical cognition in brain-damaged individuals, namely spatial neglect and impairment of working memory (Malhotra et al. 2005; Zorzi et al. 2006; Aiello et al. 2012).

Here, we provide the first demonstration of a systematic bidirectional modulation of numerical magnitude toward either lower or higher numbers. This only occurred during those stimulation conditions that induced interhemispheric conflict (i.e., combining BR with either right-sided cold caloric vestibular irrigation or left-sided warm caloric vestibular stimulation, respectively) (Arshad, Nigmatullina, Bhrugubanda et al. 2013; Arshad, Nigmatullina, and Bronstein 2013).

Indeed, the absence of any significant numerical modulation following either vestibular or visual stimulation alone compared with baseline and, critically, during the “no conflict” conditions (i.e., RIGHTWARM + RIV and LEFTCOLD + RIV conditions, see schematic in Figure 4), rules out the possibility that numerical biases were secondary to generalized arousal effects, dizziness, or visuo-vestibular mismatch (Arshad, Nigmatullina, Bhrugubanda et al. 2013). Further, substituting the BR with a visuospatial working memory task (see Supplementary Material 3) demonstrates that these effects are not specific to BR per se, but rather reflect a generalized involvement of the right lateralized fronto-parietal visuospatial attentional network (Lumer et al. 1998; Miller et al. 2000; Corbetta and Shulman 2002; Arshad, Nigmatullina, and Bronstein 2013). Critically, as right-sided cold and left-sided warm water irrigations both elicit left-beating vestibular nystagmus (Cawthorne et al. 1942; Fitzgerald and Hallpike 1942; Barnes 1995); it was found that when these irrigations were combined with visual stimulation, it modulated numerical magnitude in opposing directions. Hence, eye movements can also be ruled out as the cause of the observed numerical biasing (Loetscher et al. 2010).

Accordingly, the results from Experiments 1, 2, and 3 demonstrate that the numerical biases observed following our physiological manipulations resulted from relative unihemispheric inhibition. That is, during the LEFTWARM + RIV condition, there is a left hemisphere-predominant response with associated right hemisphere inhibition and subsequent biasing toward larger numbers. This is in keeping with the observations that pathological biases toward higher numbers occur during large interval number pair bisection tasks (as implemented herein), following lesions that result in a rightward attentional bias (Zorzi et al. 2002, 2006). In contrast, during RIGHTCOLD + RIV, there is left hemisphere inhibition following interhemispheric conflict, resulting in biasing of numerical judgements toward smaller numbers. This account is additionally corroborated by our computational model, which suggests that numerical allocation is subject to dynamic interhemispheric competition and predicts not only the results of our stimulation paradigm but also those of the SNARC effect (see Supplementary Material 6) (Dehaene et al. 1993).

Given that previous reports have demonstrated a close link between spatial attention and numerical control mechanisms (Dehaene et al. 1993; Zorzi et al. 2002; Umiltà et al. 2009), one possible account for our findings is that they are secondary to shifts in spatial attention (Fischer et al. 2003). Indeed, our data from the number pair bisection task is in line with the vast majority of previous studies in numerical cognition, in that it appears to suggest an inherent link between number and space (Hubbard et al. 2005; Stoianov et al. 2008; Umiltà et al. 2009). However, to directly probe numerical–spatial interactions, we asked subjects to reproduce culturally neutral clock faces. In the conventional representation of both clock faces and the MNL, there is an inherent left and right side, but importantly, numbers in each are mapped on to the opposite sides of space. That is, in the MNL, small numbers are found on the left side of space, whereas on a clock face, smaller numbers are represented on the right side of space (Aiello et al. 2012).

Intriguingly, we observed that the distortions of the clock drawings were in the “opposite” direction to those observed during the number pair bisection task. Hence, the results of the clock drawings provide strong evidence that the numerical effects observed are not directly linked to a spatially lateralized attentional bias for 3 main reasons. First, the lateral displacements that we observed followed a directional bias opposite to that which would be expected from a spatially lateralized effect following the relative inhibition of each hemisphere (Kinsbourne 1977; Szczepanski et al. 2010). Secondly, the critical absence of any systematic displacement in the alphabet clock conditions demonstrates that these effects, as in the study by Aiello and colleagues, are primarily numerical in origin rather than secondary to any lateralized bias of spatial attention (Aiello et al. 2012). Thirdly, in a supplemental experiment, we observed no differences in straight ahead pointing ability when comparing any of the CALORIC + RIV conditions with their corresponding Caloric-only conditions (see Supplementary Material 7).

Further support for the above viewpoint stems from previous work demonstrating that spatial attention shifts following the elicitation of nystagmus can be coupled to either the slow (Rubens 1985) or fast phase (Teramoto et al. 2004; Figliozzi et al. 2005, 2010; Watanabe et al. 2011) component of the eye movement. The direction of the shift appears to be dependent upon the stimulus employed to elicit the nystagmic eye movement. Regardless of whether the shifts in spatial attention occur in the direction of the fast or slow phase, the fact remains that as both RIGHTCOLD + RIV and LEFTWARM + RIV conditions induce left-beating nystagmus, they were associated with numerical biasing in opposing directions. This provides further direct support for a dissociation between numerical and spatial biases; however, our results are in apparent contrast to those of a recent study that employed passive whole-body vestibular stimulation (Hartmann et al. 2012). Namely, Hartmann and colleagues demonstrated a bidirectional relationship between the generation and processing of numerical magnitude and self-motion detection, supporting the view that a close relationship exists between spatial attention and numerical control mechanisms (Dehaene et al. 1993; Zorzi et al. 2002; Umiltà et al. 2009). We propose that these opposing findings are due to the fact that numerical–spatial links are much more likely to be generated when the task requires left-to-right coding of motor responses, as opposed to purely verbal responses that do not require directional specific motor coding (Rotondaro et al. 2015).

The notion that at least some degree of dissociation in certain circumstances can exist between numerical magnitude and spatial attention mechanisms has been hinted at in previous findings from right brain-damaged individuals (Doricchi et al. 2005; van Dijck et al. 2011; Aiello et al. 2012). Indeed, such dissociation has recently been demonstrated in a study where numerosity was shown to be topographically mapped in the parietal lobe, but critically with no relationship to visuospatial responses (Harvey et al. 2013). However, it has been argued that this finding was potentially confounded due to variability introduced by nonnumerical sensory cues associated with numerosity (Gebuis et al. 2014). We observed that in the condition that resulted in preferential activation of the right hemisphere (i.e., RIGHTCOLD + RIV), the numerical clocks showed an expansion for the spatial representation devoted to smaller numbers (i.e., increased spacing between these numbers) and compression of space between larger numbers. The converse was found for numerical clock drawings during preferential activation of the left hemisphere (i.e., LEFTWARM + RIV). That is, we observed increased spacing between larger numbers and compression of space between smaller numbers. Note that individual differences in inter-digit spacing were not related to hand dominance (see Supplementary Material 5). Thus, our results, using Arabic notated numerical magnitude and hence avoiding the associated confound of nonnumerical sensory cues, provide the first demonstration that numerical magnitude is topographically mapped at the cortical level.

Taken together, our data provide the underpinnings of a coherent model to explain numerical magnitude allocation in the human brain. Our findings demonstrate that the right hemisphere is disproportionately responsible for the allocation of smaller numbers, suggestive of a cortical magnification factor. We propose that the MNL can be equated to context-dependent encoding of small numbers in association with the left side of space through disproportionate representation in the right hemisphere, with larger numbers being represented in association with rightward space in the left hemisphere. Because of this lateralization of numerical encoding and the similarity of the mechanisms underpinning numerical allocation as well as spatial attention, under most circumstances smaller numbers are associated with the left side of space and larger numbers with the right side. However, in specific experimental conditions, there can be dissociation between number size and side of space. Hemispheric numerical magnitude allocation appears to be continually updated in a relative manner, rather than inherently associated with a particular hemisphere.

To conclude, using a multi-method approach in neurologically intact individuals, we provide the first demonstration of a bidirectional modulation of numerical magnitude and have demonstrated the pivotal role of dynamic interhemispheric competition for numerical allocation and representation in the human brain. Our suggested model not only provides a clear account for our results, but also predicts previous key findings in the field, opening the way for future studies to further explore the relationship between interhemispheric interactions and number allocation.

## Authors’ Contributions

Q.A. conceptualized study; Q.A., Y.N., A.M.B., and P.A.M. designed the experiments. Q.A. and P.A. performed the number pair bisection and clock drawing (i.e., motor transformation) experiments. Q.A., S.S., and U.G. performed the tDCS number experiment. R.E.R., P.A., Y.N., S.S., and U.G. performed analysis and statistics for visuo-motor transformation task. Q.A., S.K., K.S., and S.S. performed the tDCS eye movement experiment. Y.N. performed analysis and statistics for MNL bisection task. R.N. performed computational modeling. Y.N. prepared all figures for the manuscript. Q.A. and P.M. wrote the manuscript. Y.N., U.G., R.E.R., R.C.K., and A.M.B. edited the manuscript; R.C.K. provided critical input and expertise in analysis and interpretations of the results. A.M.B. and P.A.M. supervised the project.

## Supplementary Material

Supplementary material can be found at: http://www.cercor.oxfordjournals.org/.

## Funding

A.M.B. is funded by the UK Medical Research Council (UK) and P.A.M. receives research funding from the Higher Education Council for England. This research was supported by the National Institute for Health Research (NIHR) Imperial Biomedical Research Centre. Funding to pay the Open Access publication charges for this article was provided by Research Council UK Funding.

## Supplementary Material

Supplementary Data
