# Supplementary material for: Bidirectional Modulation of Numerical Magnitude
Source: Cereb Cortex. 2016 Feb 14;26(5):2311–24. doi: 10.1093/cercor/bhv344 (PMC4830300; doi:10.1093/cercor/bhv344)
Supplement: Supplementary Data [file supp_26_5_2311__index.html]

Bidirectional Modulation of Numerical Magnitude — Supplementary Data 

# Bidirectional Modulation of Numerical Magnitude

## Supplementary Data

Supplementary Data

- Supplementary Data - Docx file
