# Supplementary material for: Bidirectional Modulation of Numerical Magnitude
Source: Cereb Cortex. 2016 Feb 14;26(5):2311–24. doi: 10.1093/cercor/bhv344 (PMC4830300; doi:10.1093/cercor/bhv344)
Supplement: Supplementary Data [file supp_bhv344_bhv344supp.docx]

**Bidirectional modulation of numerical magnitude**

**Running Title: Control of Number Allocation**

**SUPPLEMENTAL DATA**

**Supplemental material 1; Analysis of clock drawings**

Two different methods were employed to analyse the clock drawings: Centre of Mass and Number spacing. For illustration purposes only, we performed heat map analysis.

1. Centre of Mass analysis*:* To analyse the behavioural data from the clock drawing experiment we developed an objective process for measuring the relative horizontal distortion in each image. This was based on the position of the COM of the drawing relative to a reference point. As each clock was drawn starting with the numeral 12, we used the position of the 12 as the internal reference point for each image. Since the clock drawings differed between individuals, we compared the position of the COM of the whole drawing relative to the position of the reference point with respect to the size of the image canvas. This ensured that the position of the COM of the drawing could be calculated as a fraction of the size of the image canvas for each clock, providing a scale invariant measure of distortion for each participant. There were two critical steps in pre-processing the images. First, the 12 was aligned with the horizontal midpoint of the *image canvas.* This was achieved by calculating the COM of the 12, and then shifting the drawing laterally on the canvas such that it was aligned with the horizontal midpoint of the image canvas. The image canvas was then cropped so that the most lateral element of the drawing was within 2 pixels of the edge of the image canvas. We then calculated shifts in the COM of the drawing relative to the size of the image canvas, thus providing a scale invariant index of the position of the COM of the drawing relative to the 12. This relative distortion approach enabled within-subject comparisons to be made for the different stimulation conditions irrespective of the physical size of the clocks and was developed to be sensitive to horizontal shifts in COM. All data were digitised using a high resolution flatbed scanner (Epson 1650, 1600 dpi) and then processed using MATLAB (Mathworks) version 7.14.0.739 (R2012a). The COM was calculated using code adapted from the MATLAB file exchange (centerOfMass.m, author: Jered Wells, Duke University).
2. *Number spacing analysis*: We also explored the average spacing of numbers on the right and left sides of the clock faces. The images were prepared as described in the centre of mass analysis, and then the X and Y coordinates of the centre of mass for each number character were calculated. This was then used to generate the distance between adjacent numbers for each clock. The mean spacing between the numbers on the right side of the clock face (12->6) and those on the left hand side of clock face (6->12) was calculated and normalised by the mean separation between all characters in the clock face. Hence a single value for each clock side was produced and a ratio of the separation between numbers on the right and the numbers on the left was calculated. A ratio of >1 suggests an average expansion of spacing between numbers on the right side of the clock or contraction of spacing between numbers on the left side.

*Heat map Analysis*: To graphically illustrate the lateral shifts observed in the ‘Caloric+RIV’ conditions we used a ‘heat mapping’ technique to display these shifts for each condition (Fig. S1). We specifically were interested in comparing the relative position of the numbers in each clock and how they were spatially distributed. In addition to the pre-processing steps taken for the COM analysis, the images were transformed into a standard space such that the distance in pixels between numerals 12 and 6 was identical for all the drawings collected. This step enabled images for multiple participants to be overlapped for each condition. The images were then convolved with a 2D Gaussian kernel, generated using code adapted from the Matlab file exchange (file exchange “customgauss”, author: Thomas Diederiksen). The individual heat maps were then binarised and summated for all participants for each condition. A maximum value of 10 was given where there was maximal overlap, or 0 for no overlap between participants (Fig. S1).

**
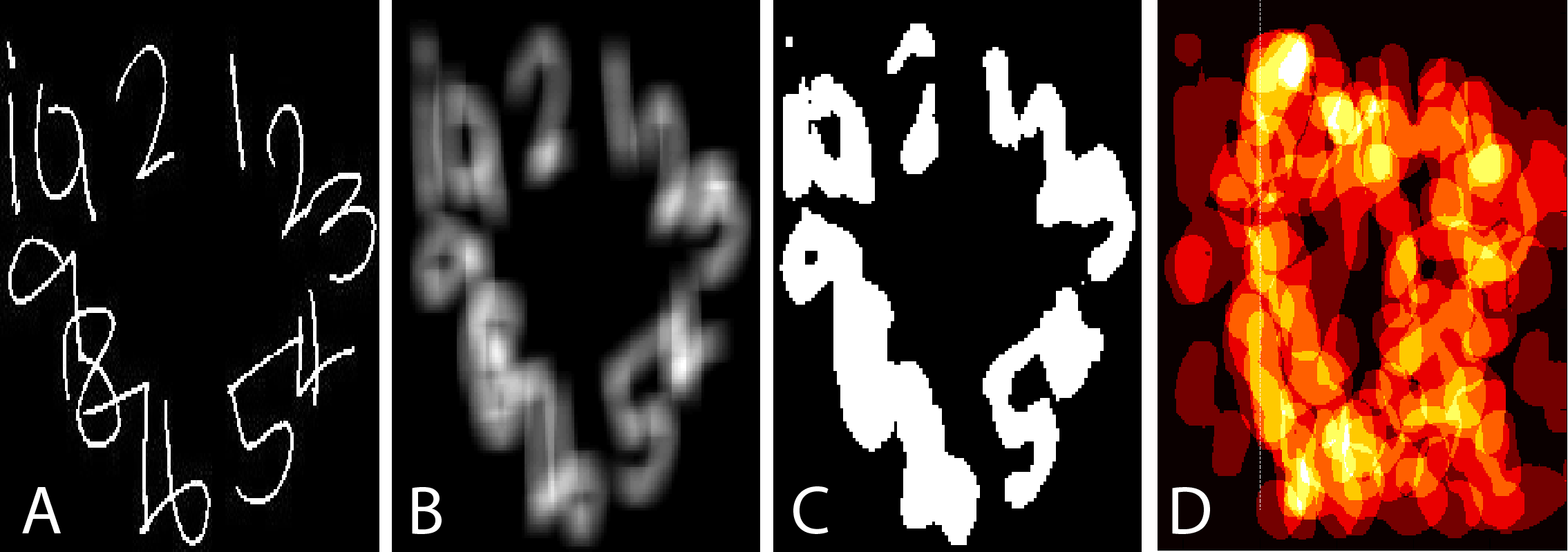
**

Figure S1; Each clock was processed prior to combining to generate heat maps for each condition. (A) Raw imported image. (B) Image convolved with 2D Gaussian. (C) Binarised image following convolution. (D) Group overlap image (n=10).

**Supplemental material 2; Representative example of numerical clock drawings**

**
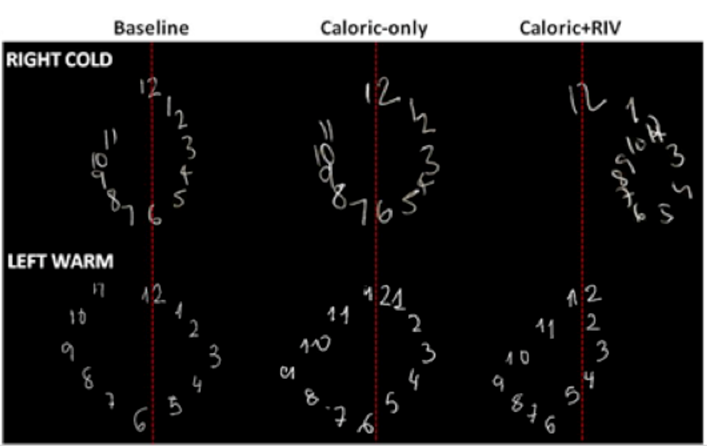
**

Figure S2; Representative numerical clock drawings from a single subject. Upper panel shows the drawings for the baseline condition (i.e. no stimulation /darkness), following RIGHTCOLD caloric only and RIGHTCOLD+RIV all drawn *clockwise.* Note the rightward lateral displacement of the numerical clock drawing following RIGHTCOLD+RIV. Lower panel shows the drawings for the for the baseline condition (darkness, no stimulation), following LEFTWARM caloric only and LEFTWARM+RIV all drawn *anti-clockwise*. Note the leftward lateral displacement of the numerical clock drawing following LEFTWARM+RIV

**Supplemental material 3; Physiological manipulation of numerical magnitude using a visuo-spatial working memory task and vestibular stimulation**

Previous reports have demonstrated that vestibular activation alters the switching rate of alternate percepts during the experience of binocular rivalry (Miller et al. 2000). To demonstrate that the effects reported in the main experiments were not specific to binocular rivalry per se, but rather secondary to a generalised involvement of visuospatial processing resources in a right lateralised fronto-parietal attention network (Knapen et al. 2011; Lumer, Friston, Rees 1998; Paffen and Van der Stigchel 2010; Sterzer, Kleinschmidt, Rees 2009) we substituted the rivalry component of the stimulation paradigm with a visuospatial working memory task (VST) (Brooks 1967; Corbetta and Shulman 2002),which we have previously employed and shown to have comparable effects (Arshad, Nigmatullina, Bronstein 2013). This consisted of a modified Brooks visuospatial paradigm where numbers were substituted with shapes (Brooks 1967). Subjects were verbally presented and required to visually imagine a 3X3 grid and 6 shapes (e.g. circle, rectangle, and triangle) that were randomly allocated to a grid position. The subject’s task was to remember the shape and its allotted position in the grid. After the task the subjects were required to recall the shapes and their respective position. The minimum criterion set was 4 correct shapes in the allotted positions out of a total of 6 (Arshad et al. 2013; Brooks 1967).

A total of 20 right-handed subjects participated (Handedness score over 40) (Oldfield 1971) (12 female, age range 18-24 years, mean age 22 years). 10 subjects participated in cold and 10 subjects in warm water irrigations. All subjects were naive to purpose of study had no history of otological, ophthalmological, psychiatric or neurological disorders and provided written informed consent as approved by the local ethics research committee.

No effect of performing the VST alone was observed upon numerical judgements [VST vs no VST: p > 0.05, F (2,18) = 0.10, Repeated Measures ANOVA]. During combined stimulation (See Figure S3), repeated measures ANOVA (3x2x2) [within-subjects factors: condition; 3 levels (BASELINE, CALORIC, CALORIC+VST), side of caloric stimulation; 2 levels (right, left ear) and temperature of caloric stimulation; 2 levels (warm, cold)] revealed that both the temperature of caloric and side of caloric stimulation showed significant main effects [p < 0.01, F (1,9) = 13.4 and p < 0.05, F (1,9) = 9.73 respectively]. The following interactions were significant: temperature*side of stimulation, temperature*condition and side of stimulation*condition [p < 0.001, F (1,9) = 42.2; p = 0.023, F (2,18) = 5.01; p = 0.005, F (2,18) = 8.08 respectively]. Post-hoc paired t-tests showed no significant difference (all p > 0.05) between baseline and caloric-only conditions. Post-hoc paired t-tests with Bonferroni corrections between caloric-only and CALORIC+VST showed significant differences only for RIGHTCOLD+VST (bias towards smaller numbers) (Fig. S3A) and LEFTWARM+VST (bias towards larger numbers) [p = 0.004, t = 5.12; p = 0.012, t = 3.98 respectively] (Fig. S3B). No significant differences were observed for either LEFTCOLD+VST or RIGHTWARM+VST (p > 0.05) (Fig; S3A and S3B).

Figure S3 Results from mental number pair bisection experiments following physiological manipulations. We present the mean % bisection error from the midpoint of the numerical interval. (A) ‘Caloric+VST’ condition (grey diamonds) resulted in subjects significantly underestimating the midpoint (i.e. shift to the left as indicated by red arrow) when compared to ‘Caloric-only’ (black diamonds) condition following RIGHTCOLD+VST (lower left hand side panel), but no effect was found during LEFTCOLD+VST (upper left hand side panel). (B) In contrast, following LEFTWARM+VST (upper right hand side panel) the subjects showed significant shift towards larger numbers (i.e. rightward shift as indicated by red arrow), suggesting overestimation of the midpoint (upper panel). No effect of RIGHTWARM+VST was observed (lower right hand side panel). Grey shaded area in panels indicates 95% confidence limits calculated from baseline measures (i.e. no caloric or vestibular stimulation). Dashed line at 0 corresponds 0% error i.e. accurate bisection. Data marked ** is significant at p < 0.01; data marked * is significant at p < 0.05. Error bars indicate standard errors.

**Supplemental material 4; Effects of tDCS alone upon number pair bisection**

**
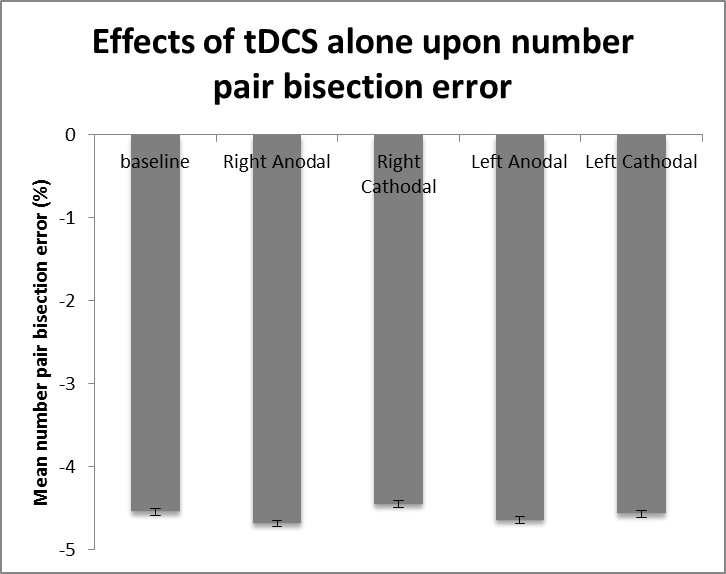
**

Figure S4 Results from mental number pair bisection task following application of unipolar frontal tDCS alone. On the x axis we represent the 5 different conditions. On the y axis we represent the mean number pair bisection error (%). As shown, none of the four tDCS conditions (i.e. right anodal, right cathodal, left anodal or left cathodal) modulated the numerical responses compared to the baseline. Error bars indicate standard error.

**Supplemental material 5; Analysis of inter-digit spacing during clock drawings**

For the numerical clock drawings we performed the inter digit spacing analysis as described above for all caloric only conditions and CALORIC+RIV conditions. As shown in Figure S4 below, the inter-digit spacing falls outside the 95% confidence intervals only for RIGHTCOLD+RIV and LEFTWARM+RIV conditions. Following RIGHTCOLD+RIV we observed an expansion for smaller numbers (blue circles) and compression for larger numbers (red squares). Following the LEFTWARM+RIV condition we observed compression for small numbers (blue circle) and expansion of larger numbers (red squares). To ascertain whether these changes in inter-digit spacing were related to hand dominance we correlated each individual’s handedness score as determined by the Edinburgh handedness inventory questionnaire with individual differences in inter-digit spacing. For RIGHTCOLD+RIV, we observed no correlation between handedness and expansion between smaller numbers (R^2^ 0.014 p > 0.05 Pearson’s correlation) nor for compression between larger numbers (R^2^ 0.037 p > 0.05 Pearson’s correlation). For LEFTWARM+RIV, we observed no correlation between handedness and expansion between large numbers (R^2^ 0.124 p > 0.05 Pearson’s correlation) nor the compression between smaller numbers (R^2^ 0.0758 p > 0.05 Pearson’s correlation).

**
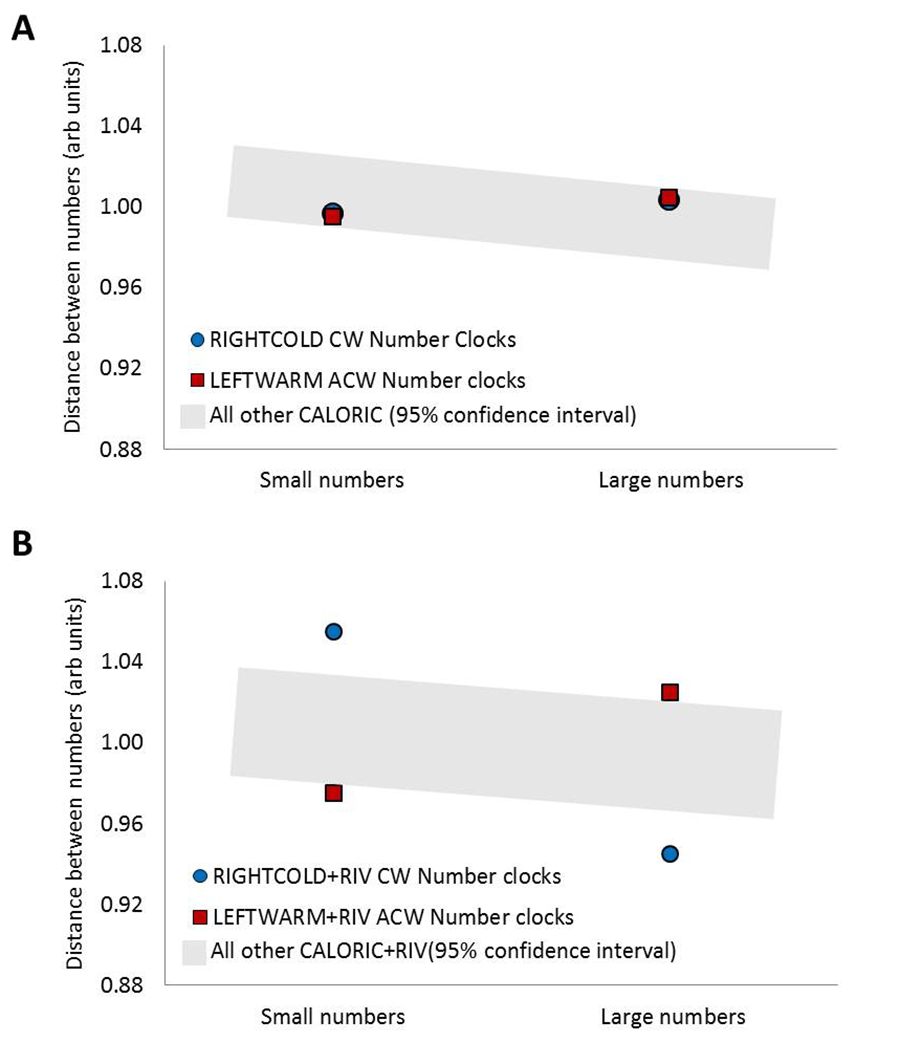
**

Figure S5 Showing inter digit spacing for all the numerical clocks drawn during the caloric only conditions (A) and CALORIC+RIV conditions (B). Grey shaded area in both panels indicates 95% confidence limits with respect to the distance between numbers. In (A) the distance between numbers for both right cold caloric (blue circle) and left warm caloric (red square) alone, which as shown falls within the 95% confidence intervals. As shown in (B), the inter-digit spacing falls outside the 95% confidence intervals only for RIGHTCOLD+RIV (blue circles show for expansion for smaller and compression for larger numbers) and LEFTWARM+RIV (red squares show compression for small numbers and expansion of larger numbers) conditions.

**Supplemental material 6; Verification of the computational model by applying it to the SNARC effect**

Verification of our computational model is provided by applying it to the SNARC effect. The SNARC effect demonstrates that subjects respond quicker with the right hand if the number is larger than the reference and quicker with the left hand if the number is smaller than the reference (Dehaene, Bossini, Giraux 1993). It is proposed that larger/left and smaller/right are conflicting pathways whereas smaller/left and larger/right do not introduce conflict. More errors are made if the task involves a conflicting pathway which can be modelled as follows. Let $p_{<}$ and $p_{>}$ be the probabilities that the subject produces the answer that corresponds to the presented number being smaller and, respectively, larger than the reference. Let $s_{1}$ be the variable denoting the state of the response; $s_{1}=1$response is “greater” and $s_{1}=-1$ response is “smaller”. Let $s_{2}$ be the variable denoting the nature of the task; $s_{2}=1$ respond with right hand and $s_{2}=-1$ respond with left hand. The appropriate energy function for the SNARC experiments is;

$$E\left( s_{1},s_{2},J,h \right)=-hs_{1}-Jhs_{1}s_{2}, [1]$$

where $h>0$and $J>0$.

The probabilities are given by the Boltzmann distribution:

$$p_{>}\left( s_{2},J,h \right)=\frac{\exp\left( E\left( 1,s_{2},J,h \right) \right)}{\exp\left( E\left( 1,s_{2},J,h \right) \right)+\exp\left( E\left( -1,s_{2},J,h \right) \right)} [2]$$

$$p_{<}\left( s_{2},J,h \right)=\frac{\exp\left( E\left( -1,s_{2},J,h \right) \right)}{\exp\left( E\left( 1,s_{2},J,h \right) \right)+\exp\left( E\left( -1,s_{2},J,h \right) \right)} [3]$$

Parameter $h$ denotes the magnitude of the difference between the presented and the reference number. If the two numbers are equal, $h=0,$and the response is equally likely to be either “larger” or “smaller”. Parameter $J$denotes the strength of the conflict.

The first term in equation [1] simply states that increasing the difference between the given number and the reference number increases the probability of the right answer. The second term in equation [2] is the conflict term implemented to reduce the probability of the right answer if the task contains a conflict and to increase the probability of the right answer in the case of a non-conflict task. Figure S5 shows the plot of the function $p_{>}$ as a function of $hs_{1}$ for three different values of $Js_{2}$. Thus the SNARC effect and our model can be explained by the conflict term $-Jhs_{1}s_{2}$ of the energy function [1].


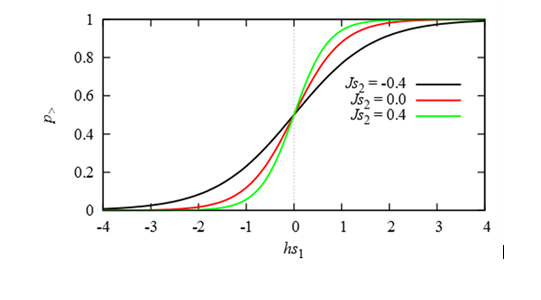


Figure S6; Computational modelling. Here, we apply the model to predict the SNARC effect specifically the aspect of providing the answer “greater” than the midpoint as a function of the difference between the given number and the reference number for three different conditions. The curve of $Js_{2}=0$is a control i.e. a curve representing no conflict, for example, when the subject is asked to verbally respond to the magnitude comparison task rather than with a hand. We can observe that when $s_{2}<0$ (the subject has to respond with the left hand), then the probability of the right answer, when the given number is greater decreases, and when the given number is smaller increases. Conversely, when $s_{2}>0$ (the subject has to respond with the right hand), then the probability of the right answer when the given number is greater increases, and when the given number is smaller decreases.

**Supplemental material 7; Straight ahead pointing experiment**

In order to directly ascertain whether the observed modulation in the visuo-motor transformations were attributable solely to a numerical bias and not a spatially lateralised motor bias we performed a straight-ahead pointing experiment.  The protocol was adapted from a study that examined the relationship between subjective straight-ahead and long line bisection in neglect patients. 10 new right handed healthy subjects (7 males /3 females; age range 20-30, mean age 24 years) were recruited. The same experimental set-up was used as the one for visuo-motor transformation task.  An A2 sized drawing board fixed with white A3 paper was placed on the lap against the subject’s knees.  In the baseline condition (darkness), subjects were asked to raise their right hand, holding the pen, so that the experimenter could guide the pen to the ‘start’ point on the marked A3 paper by holding the pen without touching the subject’s hands.  Subjects were then instructed to mark a new point straight ahead, approximately 30cm from the start point. This mark was taken as their subjective straight ahead (SSA) (Richard et al. 2004).  This was performed for both caloric-only and Caloric+RIV conditions. In each of the conditions, both left and right ear irrigations with cold and warm caloric irrigations were performed in a randomized order. Five trials were carried out in each condition. No significance for factor ‘side’ (p>0.05, F=2.26,df=1) nor for condition (p>0.05, F=3.08,df=2) was observed, with either cold or warm caloric alone compared to the corresponding CALORIC+RIV condition.

References

Arshad Q, Nigmatullina Y, Bronstein AM. 2013. Handedness-related cortical modulation of the vestibular-ocular reflex. J Neurosci 33(7):3221-7.

Arshad Q, Nigmatullina Y, Bhrugubanda V, Asavarut P, Obrocki P, Bronstein AM, Roberts RE. 2013. Separate attentional components modulate early visual cortex excitability. Cortex 49(10):2938-40.

Brooks LR. 1967. The suppression of visualization by reading. Q J Exp Psychol 19(4):289-99.

Corbetta M and Shulman GL. 2002. Control of goal-directed and stimulus-driven attention in the brain. Nature Reviews Neuroscience 3(3):201-15.

Dehaene S, Bossini S, Giraux P. 1993. The mental representation of parity and number magnitude. J Exp Psychol : Gen 122(3):371.

Knapen T, Brascamp J, Pearson J, van Ee R, Blake R. 2011. The role of frontal and parietal brain areas in bistable perception. J Neurosci 31(28):10293-301.

Lumer ED, Friston KJ, Rees G. 1998. Neural correlates of perceptual rivalry in the human brain. Science 280(5371):1930-4.

Miller SM, Liu GB, Ngo TT, Hooper G, Riek S, Carson RG, Pettigrew JD. 2000. Interhemispheric switching mediates perceptual rivalry. Current Biology 10(7):383-92.

Oldfield RC. 1971. The assessment and analysis of handedness: The edinburgh inventory. Neuropsychologia 9(1):97-113.

Paffen CL and Van der Stigchel S. 2010. Shifting spatial attention makes you flip: Exogenous visual attention triggers perceptual alternations during binocular rivalry. Attention, Perception, & Psychophysics 72(5):1237-43.

Richard C, Rousseaux M, Saj A, Honore J. 2004. Straight ahead in spatial neglect: Evidence that space is shifted, not rotated. Neurology 63(11):2136-8.

Sterzer P, Kleinschmidt A, Rees G. 2009. The neural bases of multistable perception. Trends Cogn Sci (Regul Ed ) 13(7):310-8.
